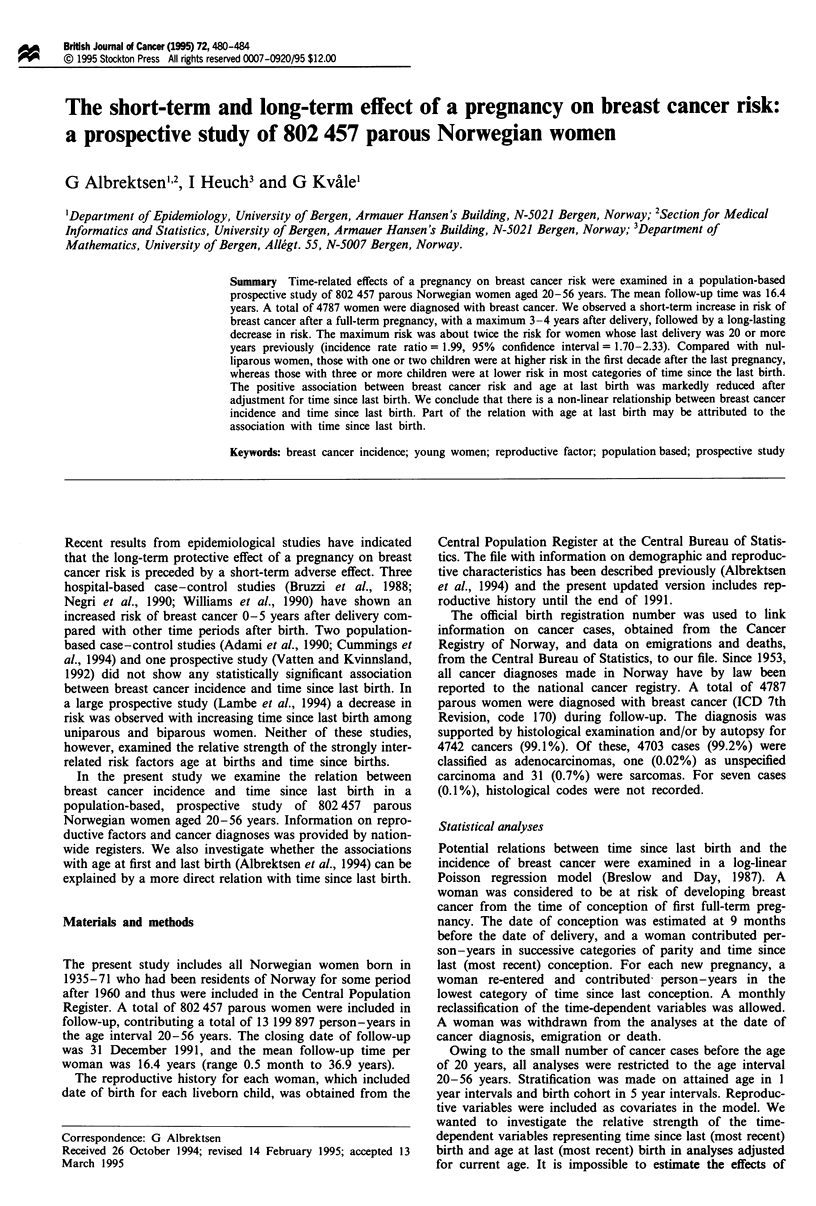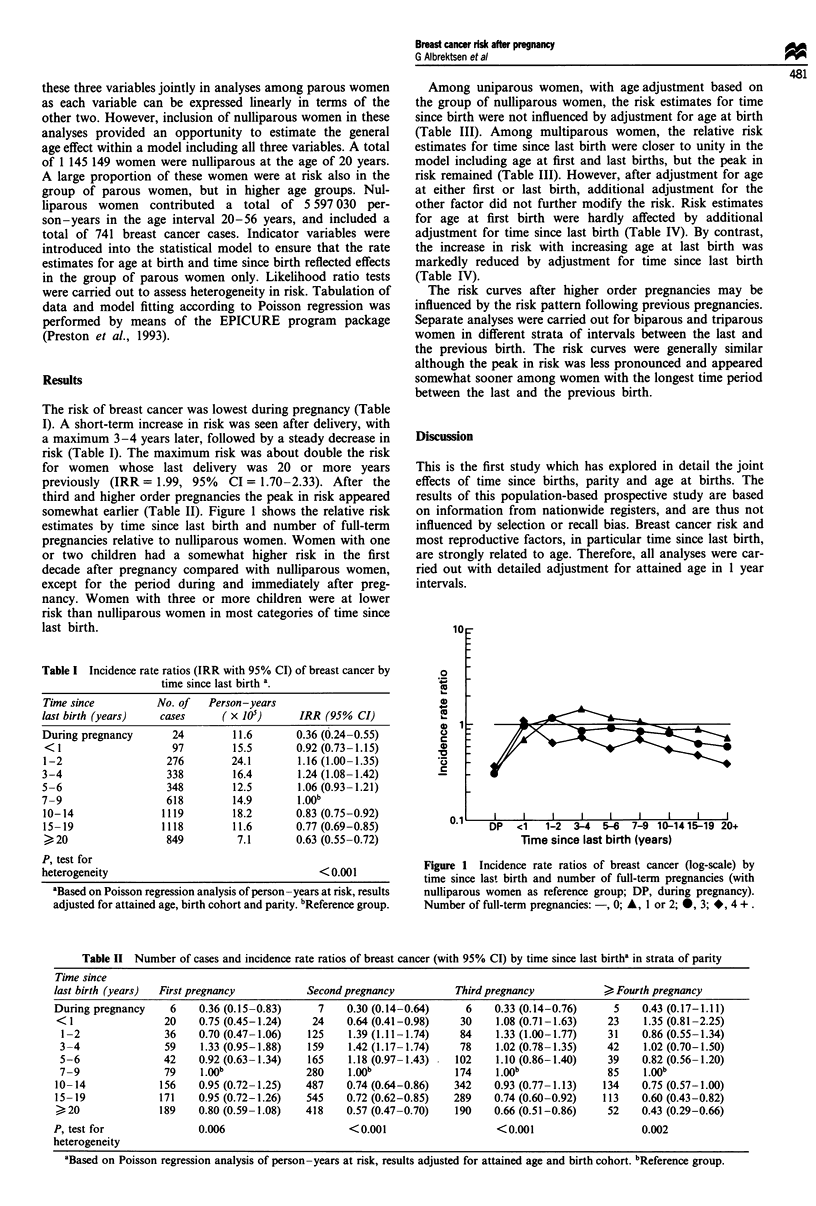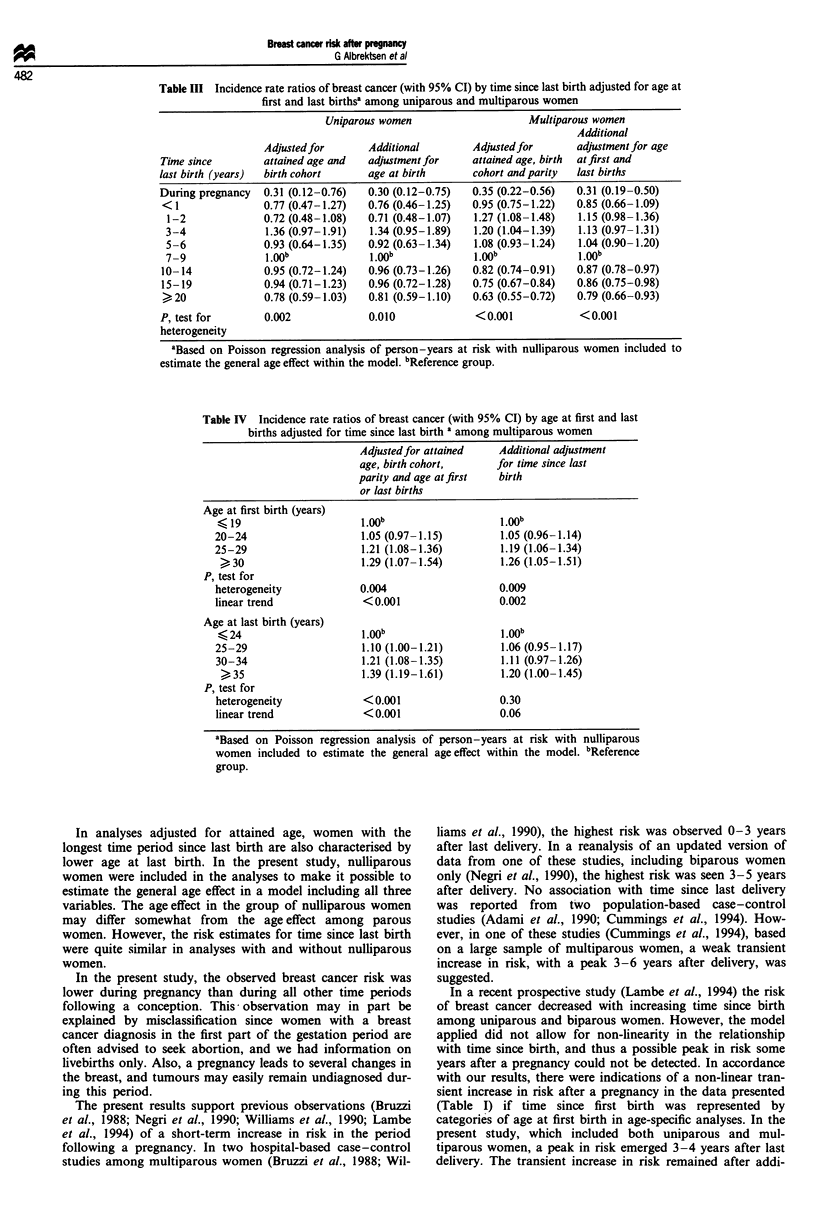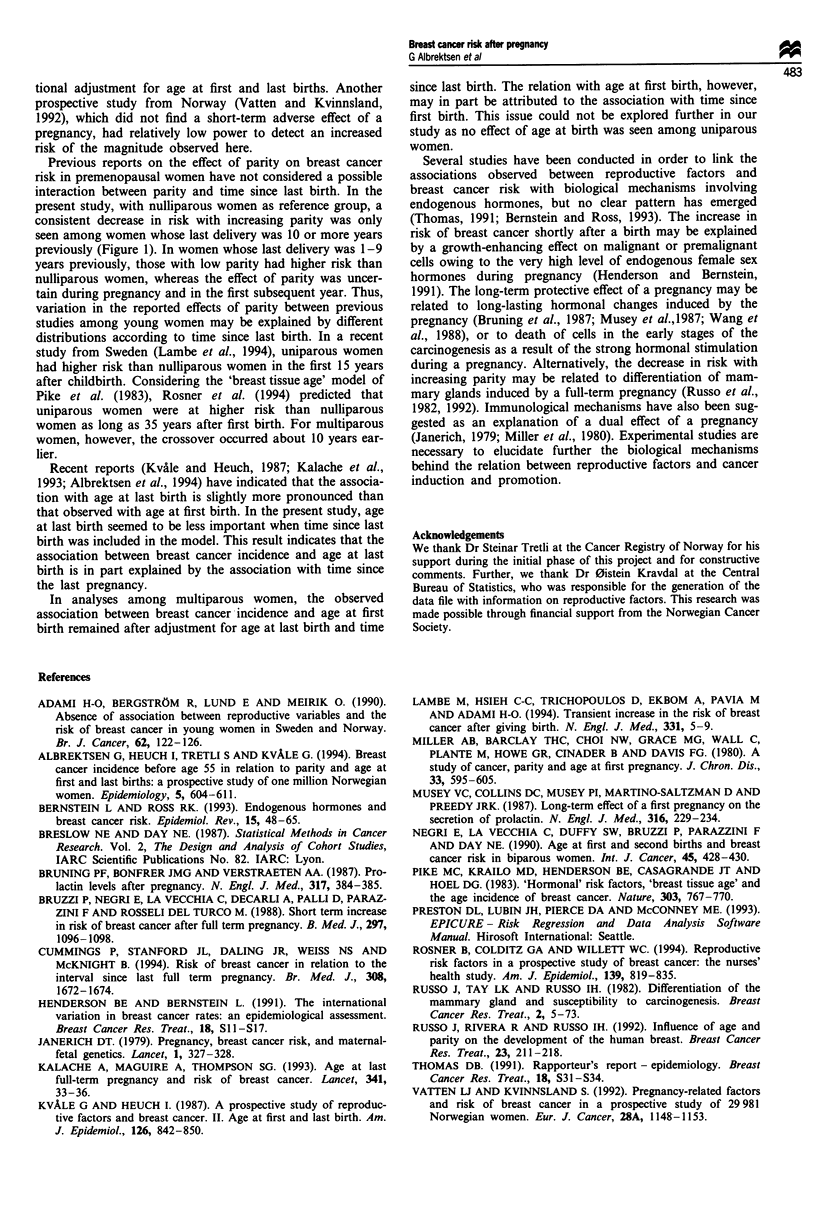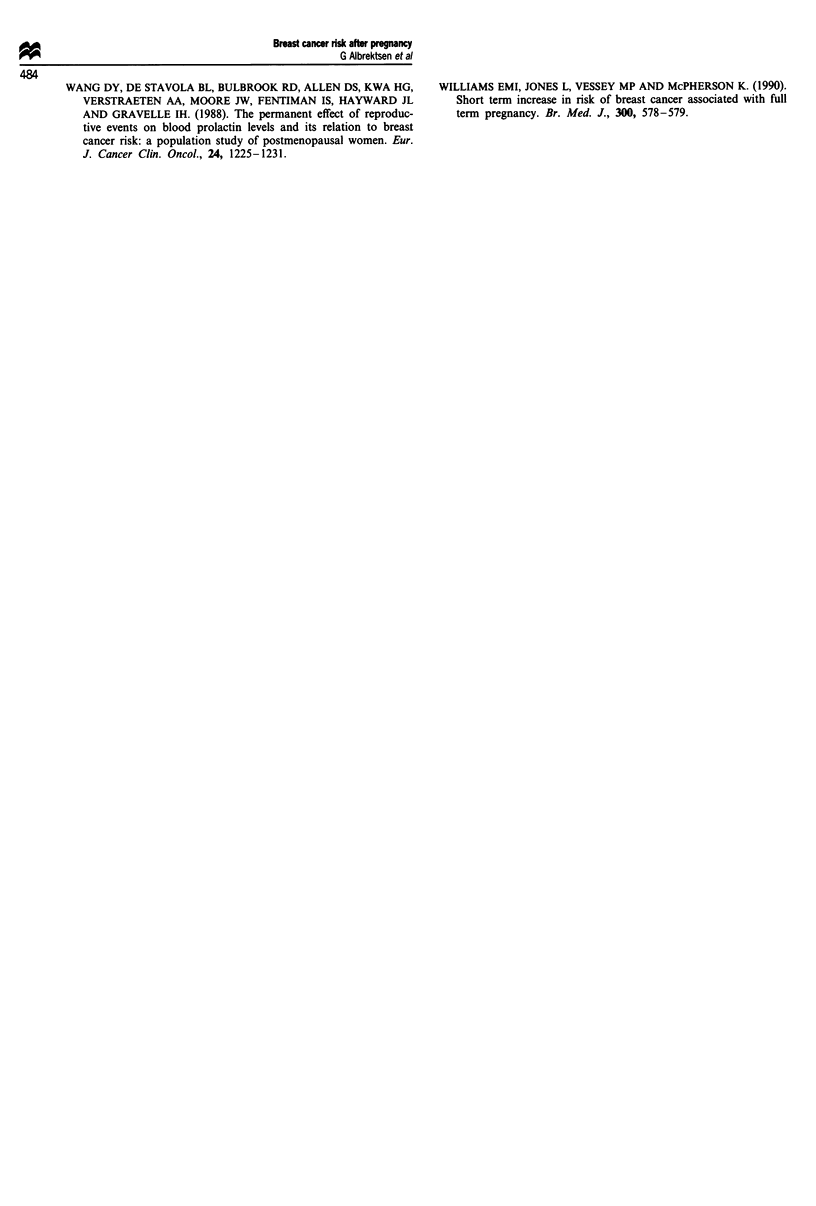# The short-term and long-term effect of a pregnancy on breast cancer risk: a prospective study of 802,457 parous Norwegian women.

**DOI:** 10.1038/bjc.1995.359

**Published:** 1995-08

**Authors:** G. Albrektsen, I. Heuch, G. Kvåle

**Affiliations:** Department of Epidemiology, University of Bergen, Norway.

## Abstract

Time-related effects of a pregnancy on breast cancer risk were examined in a population-based prospective study of 802,457 parous Norwegian women aged 20-56 years. The mean follow-up time was 16.4 years. A total of 4787 women were diagnosed with breast cancer. We observed a short-term increase in risk of breast cancer after a full-term pregnancy, with a maximum 3-4 years after delivery, followed by a long-lasting decrease in risk. The maximum risk was about twice the risk for women whose last delivery was 20 or more years previously (incidence rate ratio = 1.99, 95% confidence interval = 1.70-2.33). Compared with nulliparous women, those with one or two children were at higher risk in the first decade after the last pregnancy, whereas those with three or more children were at lower risk in most categories of time since the last birth. The positive association between breast cancer risk and age at last birth was markedly reduced after adjustment for time since last birth. We conclude that there is a non-linear relationship between breast cancer incidence and time since last birth. Part of the relation with age at last birth may be attributed to the association with time since last birth.


British Journal of Cancer (1995) 72, 480–484
© 1995 Stockton Press All rights reserved 0007–0920/95 $12.00

### The short-term and long-term effect of a pregnancy on breast cancer risk: a prospective study of 802 457 parous Norwegian women

G Albrektsen[1,2], I Heuch[3] and G Kvåle[1]

[1]*Department of Epidemiology, University of Bergen, Armauer Hansen's Building, N-5021 Bergen, Norway;* [2]*Section for Medical Informatics and Statistics, University of Bergen, Armauer Hansen's Building, N-5021 Bergen, Norway;* [3]*Department of Mathematics, University of Bergen, Allégt. 55, N-5007 Bergen, Norway.*

**Summary** Time-related effects of a pregnancy on breast cancer risk were examined in a population-based prospective study of 802 457 parous Norwegian women aged 20–56 years. The mean follow-up time was 16.4 years. A total of 4787 women were diagnosed with breast cancer. We observed a short-term increase in risk of breast cancer after a full-term pregnancy, with a maximum 3–4 years after delivery, followed by a long-lasting decrease in risk. The maximum risk was about twice the risk for women whose last delivery was 20 or more years previously (incidence rate ratio = 1.99, 95% confidence interval = 1.70–2.33). Compared with nulliparous women, those with one or two children were at higher risk in the first decade after the last pregnancy, whereas those with three or more children were at lower risk in most categories of time since the last birth. The positive association between breast cancer risk and age at last birth was markedly reduced after adjustment for time since last birth. We conclude that there is a non-linear relationship between breast cancer incidence and time since last birth. Part of the relation with age at last birth may be attributed to the association with time since last birth.

**Keywords:** breast cancer incidence; young women; reproductive factor; population based; prospective study

Recent results from epidemiological studies have indicated that the long-term protective effect of a pregnancy on breast cancer risk is preceded by a short-term adverse effect. Three hospital-based case–control studies (Bruzzi *et al.*, 1988; Negri *et al.*, 1990; Williams *et al.*, 1990) have shown an increased risk of breast cancer 0–5 years after delivery compared with other time periods after birth. Two population-based case–control studies (Adami *et al.*, 1990; Cummings *et al.*, 1994) and one prospective study (Vatten and Kvinnsland, 1992) did not show any statistically significant association between breast cancer incidence and time since last birth. In a large prospective study (Lambe *et al.*, 1994) a decrease in risk was observed with increasing time since last birth among uniparous and biparous women. Neither of these studies, however, examined the relative strength of the strongly interrelated risk factors age at births and time since births.

In the present study we examine the relation between breast cancer incidence and time since last birth in a population-based, prospective study of 802 457 parous Norwegian women aged 20–56 years. Information on reproductive factors and cancer diagnoses was provided by nationwide registers. We also investigate whether the associations with age at first and last birth (Albrektsen *et al.*, 1994) can be explained by a more direct relation with time since last birth.

### Materials and methods

The present study includes all Norwegian women born in 1935–71 who had been residents of Norway for some period after 1960 and thus were included in the Central Population Register. A total of 802 457 parous women were included in follow-up, contributing a total of 13 199 897 person–years in the age interval 20–56 years. The closing date of follow-up was 31 December 1991, and the mean follow-up time per woman was 16.4 years (range 0.5 month to 36.9 years).

The reproductive history for each woman, which included date of birth for each liveborn child, was obtained from the Central Population Register at the Central Bureau of Statistics. The file with information on demographic and reproductive characteristics has been described previously (Albrektsen *et al.*, 1994) and the present updated version includes reproductive history until the end of 1991.

The official birth registration number was used to link information on cancer cases, obtained from the Cancer Registry of Norway, and data on emigrations and deaths, from the Central Bureau of Statistics, to our file. Since 1953, all cancer diagnoses made in Norway have by law been reported to the national cancer registry. A total of 4787 parous women were diagnosed with breast cancer (ICD 7th Revision, code 170) during follow-up. The diagnosis was supported by histological examination and/or by autopsy for 4742 cancers (99.1%). Of these, 4703 cases (99.2%) were classified as adenocarcinomas, one (0.02%) as unspecified carcinoma and 31 (0.7%) were sarcomas. For seven cases (0.1%), histological codes were not recorded.

### *Statistical analyses*

Potential relations between time since last birth and the incidence of breast cancer were examined in a log-linear Poisson regression model (Breslow and Day, 1987). A woman was considered to be at risk of developing breast cancer from the time of conception of first full-term pregnancy. The date of conception was estimated at 9 months before the date of delivery, and a woman contributed person–years in successive categories of parity and time since last (most recent) conception. For each new pregnancy, a woman re-entered and contributed person–years in the lowest category of time since last conception. A monthly reclassification of the time-dependent variables was allowed. A woman was withdrawn from the analyses at the date of cancer diagnosis, emigration or death.

Owing to the small number of cancer cases before the age of 20 years, all analyses were restricted to the age interval 20–56 years. Stratification was made on attained age in 1 year intervals and birth cohort in 5 year intervals. Reproductive variables were included as covariates in the model. We wanted to investigate the relative strength of the time-dependent variables representing time since last (most recent) birth and age at last (most recent) birth in analyses adjusted for current age. It is impossible to estimate the effects of

Correspondence: G Albrektsen
Received 26 October 1994; revised 14 February 1995; accepted 13 March 1995

these three variables jointly in analyses among parous women as each variable can be expressed linearly in terms of the other two. However, inclusion of nulliparous women in these analyses provided an opportunity to estimate the general age effect within a model including all three variables. A total of 1 145 149 women were nulliparous at the age of 20 years. A large proportion of these women were at risk also in the group of parous women, but in higher age groups. Nulliparous women contributed a total of 5 597 030 person-years in the age interval 20–56 years, and included a total of 741 breast cancer cases. Indicator variables were introduced into the statistical model to ensure that the rate estimates for age at birth and time since birth reflected effects in the group of parous women only. Likelihood ratio tests were carried out to assess heterogeneity in risk. Tabulation of data and model fitting according to Poisson regression was performed by means of the EPICURE program package (Preston *et al.*, 1993).

### Results

The risk of breast cancer was lowest during pregnancy (Table I). A short-term increase in risk was seen after delivery, with a maximum 3–4 years later, followed by a steady decrease in risk (Table I). The maximum risk was about double the risk for women whose last delivery was 20 or more years previously (IRR = 1.99, 95% CI = 1.70–2.33). After the third and higher order pregnancies the peak in risk appeared somewhat earlier (Table II). Figure 1 shows the relative risk estimates by time since last birth and number of full-term pregnancies relative to nulliparous women. Women with one or two children had a somewhat higher risk in the first decade after pregnancy compared with nulliparous women, except for the period during and immediately after pregnancy. Women with three or more children were at lower risk than nulliparous women in most categories of time since last birth.

**Table I** Incidence rate ratios (IRR with 95% CI) of breast cancer by time since last birth [a].

| *Time since last birth (years)* | *No. of cases* | *Person-years (× 10⁵)* | *IRR (95% CI)* |
|---|---|---|---|
| During pregnancy | 24 | 11.6 | 0.36 (0.24–0.55) |
| <1 | 97 | 15.5 | 0.92 (0.73–1.15) |
| 1–2 | 276 | 24.1 | 1.16 (1.00–1.35) |
| 3–4 | 338 | 16.4 | 1.24 (1.08–1.42) |
| 5–6 | 348 | 12.5 | 1.06 (0.93–1.21) |
| 7–9 | 618 | 14.9 | 1.00[b] |
| 10–14 | 1119 | 18.2 | 0.83 (0.75–0.92) |
| 15–19 | 1118 | 11.6 | 0.77 (0.69–0.85) |
| ≥20 | 849 | 7.1 | 0.63 (0.55–0.72) |
| *P*, test for heterogeneity | | | <0.001 |

[a]Based on Poisson regression analysis of person-years at risk, results adjusted for attained age, birth cohort and parity. [b]Reference group.

Among uniparous women, with age adjustment based on the group of nulliparous women, the risk estimates for time since birth were not influenced by adjustment for age at birth (Table III). Among multiparous women, the relative risk estimates for time since last birth were closer to unity in the model including age at first and last births, but the peak in risk remained (Table III). However, after adjustment for age at either first or last birth, additional adjustment for the other factor did not further modify the risk. Risk estimates for age at first birth were hardly affected by additional adjustment for time since last birth (Table IV). By contrast, the increase in risk with increasing age at last birth was markedly reduced by adjustment for time since last birth (Table IV).

The risk curves after higher order pregnancies may be influenced by the risk pattern following previous pregnancies. Separate analyses were carried out for biparous and triparous women in different strata of intervals between the last and the previous birth. The risk curves were generally similar although the peak in risk was less pronounced and appeared somewhat sooner among women with the longest time period between the last and the previous birth.

### Discussion

This is the first study which has explored in detail the joint effects of time since births, parity and age at births. The results of this population-based prospective study are based on information from nationwide registers, and are thus not influenced by selection or recall bias. Breast cancer risk and most reproductive factors, in particular time since last birth, are strongly related to age. Therefore, all analyses were carried out with detailed adjustment for attained age in 1 year intervals.

**Figure 1** Incidence rate ratios of breast cancer (log-scale) by time since last birth and number of full-term pregnancies (with nulliparous women as reference group; DP, during pregnancy). Number of full-term pregnancies: —, 0; ▲, 1 or 2; ●, 3; ◆, 4 +.

**Table II** Number of cases and incidence rate ratios of breast cancer (with 95% CI) by time since last birth[a] in strata of parity

| *Time since last birth (years)* | *First pregnancy* | | *Second pregnancy* | | *Third pregnancy* | | *≥ Fourth pregnancy* | |
|---|---|---|---|---|---|---|---|---|
| During pregnancy | 6 | 0.36 (0.15–0.83) | 7 | 0.30 (0.14–0.64) | 6 | 0.33 (0.14–0.76) | 5 | 0.43 (0.17–1.11) |
| <1 | 20 | 0.75 (0.45–1.24) | 24 | 0.64 (0.41–0.98) | 30 | 1.08 (0.71–1.63) | 23 | 1.35 (0.81–2.25) |
| 1–2 | 36 | 0.70 (0.47–1.06) | 125 | 1.39 (1.11–1.74) | 84 | 1.33 (1.00–1.77) | 31 | 0.86 (0.55–1.34) |
| 3–4 | 59 | 1.33 (0.95–1.88) | 159 | 1.42 (1.17–1.74) | 78 | 1.02 (0.78–1.35) | 42 | 1.02 (0.70–1.50) |
| 5–6 | 42 | 0.92 (0.63–1.34) | 165 | 1.18 (0.97–1.43) | 102 | 1.10 (0.86–1.40) | 39 | 0.82 (0.56–1.20) |
| 7–9 | 79 | 1.00[b] | 280 | 1.00[b] | 174 | 1.00[b] | 85 | 1.00[b] |
| 10–14 | 156 | 0.95 (0.72–1.25) | 487 | 0.74 (0.64–0.86) | 342 | 0.93 (0.77–1.13) | 134 | 0.75 (0.57–1.00) |
| 15–19 | 171 | 0.95 (0.72–1.26) | 545 | 0.72 (0.62–0.85) | 289 | 0.74 (0.60–0.92) | 113 | 0.60 (0.43–0.82) |
| ≥20 | 189 | 0.80 (0.59–1.08) | 418 | 0.57 (0.47–0.70) | 190 | 0.66 (0.51–0.86) | 52 | 0.43 (0.29–0.66) |
| *P*, test for heterogeneity | | 0.006 | | <0.001 | | <0.001 | | 0.002 |

[a]Based on Poisson regression analysis of person-years at risk, results adjusted for attained age and birth cohort. [b]Reference group.

**Table III** Incidence rate ratios of breast cancer (with 95% CI) by time since last birth adjusted for age at first and last births[a] among uniparous and multiparous women

| | *Uniparous women* | | *Multiparous women* | |
|---|---|---|---|---|
| *Time since last birth (years)* | *Adjusted for attained age and birth cohort* | *Additional adjustment for age at birth* | *Adjusted for attained age, birth cohort and parity* | *Additional adjustment for age at first and last births* |
| During pregnancy | 0.31 (0.12–0.76) | 0.30 (0.12–0.75) | 0.35 (0.22–0.56) | 0.31 (0.19–0.50) |
| <1 | 0.77 (0.47–1.27) | 0.76 (0.46–1.25) | 0.95 (0.75–1.22) | 0.85 (0.66–1.09) |
| 1–2 | 0.72 (0.48–1.08) | 0.71 (0.48–1.07) | 1.27 (1.08–1.48) | 1.15 (0.98–1.36) |
| 3–4 | 1.36 (0.97–1.91) | 1.34 (0.95–1.89) | 1.20 (1.04–1.39) | 1.13 (0.97–1.31) |
| 5–6 | 0.93 (0.64–1.35) | 0.92 (0.63–1.34) | 1.08 (0.93–1.24) | 1.04 (0.90–1.20) |
| 7–9 | 1.00[b] | 1.00[b] | 1.00[b] | 1.00[b] |
| 10–14 | 0.95 (0.72–1.24) | 0.96 (0.73–1.26) | 0.82 (0.74–0.91) | 0.87 (0.78–0.97) |
| 15–19 | 0.94 (0.71–1.23) | 0.96 (0.72–1.28) | 0.75 (0.67–0.84) | 0.86 (0.75–0.98) |
| ⩾20 | 0.78 (0.59–1.03) | 0.81 (0.59–1.10) | 0.63 (0.55–0.72) | 0.79 (0.66–0.93) |
| *P*, test for heterogeneity | 0.002 | 0.010 | <0.001 | <0.001 |

[a]Based on Poisson regression analysis of person–years at risk with nulliparous women included to estimate the general age effect within the model. [b]Reference group.

**Table IV** Incidence rate ratios of breast cancer (with 95% CI) by age at first and last births adjusted for time since last birth [a] among multiparous women

| | *Adjusted for attained age, birth cohort, parity and age at first or last births* | *Additional adjustment for time since last birth* |
|---|---|---|
| Age at first birth (years) | | |
| ⩽19 | 1.00[b] | 1.00[b] |
| 20–24 | 1.05 (0.97–1.15) | 1.05 (0.96–1.14) |
| 25–29 | 1.21 (1.08–1.36) | 1.19 (1.06–1.34) |
| ⩾30 | 1.29 (1.07–1.54) | 1.26 (1.05–1.51) |
| *P*, test for | | |
| heterogeneity | 0.004 | 0.009 |
| linear trend | <0.001 | 0.002 |
| Age at last birth (years) | | |
| ⩽24 | 1.00[b] | 1.00[b] |
| 25–29 | 1.10 (1.00–1.21) | 1.06 (0.95–1.17) |
| 30–34 | 1.21 (1.08–1.35) | 1.11 (0.97–1.26) |
| ⩾35 | 1.39 (1.19–1.61) | 1.20 (1.00–1.45) |
| *P*, test for | | |
| heterogeneity | <0.001 | 0.30 |
| linear trend | <0.001 | 0.06 |

[a]Based on Poisson regression analysis of person–years at risk with nulliparous women included to estimate the general age effect within the model. [b]Reference group.

In analyses adjusted for attained age, women with the longest time period since last birth are also characterised by lower age at last birth. In the present study, nulliparous women were included in the analyses to make it possible to estimate the general age effect in a model including all three variables. The age effect in the group of nulliparous women may differ somewhat from the age effect among parous women. However, the risk estimates for time since last birth were quite similar in analyses with and without nulliparous women.

In the present study, the observed breast cancer risk was lower during pregnancy than during all other time periods following a conception. This observation may in part be explained by misclassification since women with a breast cancer diagnosis in the first part of the gestation period are often advised to seek abortion, and we had information on livebirths only. Also, a pregnancy leads to several changes in the breast, and tumours may easily remain undiagnosed during this period.

The present results support previous observations (Bruzzi *et al.*, 1988; Negri *et al.*, 1990; Williams *et al.*, 1990; Lambe *et al.*, 1994) of a short-term increase in risk in the period following a pregnancy. In two hospital-based case–control studies among multiparous women (Bruzzi *et al.*, 1988; Williams *et al.*, 1990), the highest risk was observed 0–3 years after last delivery. In a reanalysis of an updated version of data from one of these studies, including biparous women only (Negri *et al.*, 1990), the highest risk was seen 3–5 years after delivery. No association with time since last delivery was reported from two population-based case–control studies (Adami *et al.*, 1990; Cummings *et al.*, 1994). However, in one of these studies (Cummings *et al.*, 1994), based on a large sample of multiparous women, a weak transient increase in risk, with a peak 3–6 years after delivery, was suggested.

In a recent prospective study (Lambe *et al.*, 1994) the risk of breast cancer decreased with increasing time since birth among uniparous and biparous women. However, the model applied did not allow for non-linearity in the relationship with time since birth, and thus a possible peak in risk some years after a pregnancy could not be detected. In accordance with our results, there were indications of a non-linear transient increase in risk after a pregnancy in the data presented (Table I) if time since first birth was represented by categories of age at first birth in age-specific analyses. In the present study, which included both uniparous and multiparous women, a peak in risk emerged 3–4 years after last delivery. The transient increase in risk remained after additional adjustment for age at first and last births. Another prospective study from Norway (Vatten and Kvinnsland, 1992), which did not find a short-term adverse effect of a pregnancy, had relatively low power to detect an increased risk of the magnitude observed here.

Previous reports on the effect of parity on breast cancer risk in premenopausal women have not considered a possible interaction between parity and time since last birth. In the present study, with nulliparous women as reference group, a consistent decrease in risk with increasing parity was only seen among women whose last delivery was 10 or more years previously (Figure 1). In women whose last delivery was 1-9 years previously, those with low parity had higher risk than nulliparous women, whereas the effect of parity was uncertain during pregnancy and in the first subsequent year. Thus, variation in the reported effects of parity between previous studies among young women may be explained by different distributions according to time since last birth. In a recent study from Sweden (Lambe *et al.*, 1994), uniparous women had higher risk than nulliparous women in the first 15 years after childbirth. Considering the 'breast tissue age' model of Pike *et al.* (1983), Rosner *et al.* (1994) predicted that uniparous women were at higher risk than nulliparous women as long as 35 years after first birth. For multiparous women, however, the crossover occurred about 10 years earlier.

Recent reports (Kvåle and Heuch, 1987; Kalache *et al.*, 1993; Albrektsen *et al.*, 1994) have indicated that the association with age at last birth is slightly more pronounced than that observed with age at first birth. In the present study, age at last birth seemed to be less important when time since last birth was included in the model. This result indicates that the association between breast cancer incidence and age at last birth is in part explained by the association with time since the last pregnancy.

In analyses among multiparous women, the observed association between breast cancer incidence and age at first birth remained after adjustment for age at last birth and time since last birth. The relation with age at first birth, however, may in part be attributed to the association with time since first birth. This issue could not be explored further in our study as no effect of age at birth was seen among uniparous women.

Several studies have been conducted in order to link the associations observed between reproductive factors and breast cancer risk with biological mechanisms involving endogenous hormones, but no clear pattern has emerged (Thomas, 1991; Bernstein and Ross, 1993). The increase in risk of breast cancer shortly after a birth may be explained by a growth-enhancing effect on malignant or premalignant cells owing to the very high level of endogenous female sex hormones during pregnancy (Henderson and Bernstein, 1991). The long-term protective effect of a pregnancy may be related to long-lasting hormonal changes induced by the pregnancy (Bruning *et al.*, 1987; Musey *et al.*,1987; Wang *et al.*, 1988), or to death of cells in the early stages of the carcinogenesis as a result of the strong hormonal stimulation during a pregnancy. Alternatively, the decrease in risk with increasing parity may be related to differentiation of mammary glands induced by a full-term pregnancy (Russo *et al.*, 1982, 1992). Immunological mechanisms have also been suggested as an explanation of a dual effect of a pregnancy (Janerich, 1979; Miller *et al.*, 1980). Experimental studies are necessary to elucidate further the biological mechanisms behind the relation between reproductive factors and cancer induction and promotion.

**Acknowledgements**
We thank Dr Steinar Tretli at the Cancer Registry of Norway for his support during the initial phase of this project and for constructive comments. Further, we thank Dr Øistein Kravdal at the Central Bureau of Statistics, who was responsible for the generation of the data file with information on reproductive factors. This research was made possible through financial support from the Norwegian Cancer Society.

### References

ADAMI H-O, BERGSTRÖM R, LUND E AND MEIRIK O. (1990). Absence of association between reproductive variables and the risk of breast cancer in young women in Sweden and Norway. *Br. J. Cancer*, **62**, 122-126.

ALBREKTSEN G, HEUCH I, TRETLI S AND KVÅLE G. (1994). Breast cancer incidence before age 55 in relation to parity and age at first and last births: a prospective study of one million Norwegian women. *Epidemiology*, **5**, 604-611.

BERNSTEIN L AND ROSS RK. (1993). Endogenous hormones and breast cancer risk. *Epidemiol. Rev.*, **15**, 48-65.

BRESLOW NE AND DAY NE. (1987). *Statistical Methods in Cancer Research*. Vol. 2, *The Design and Analysis of Cohort Studies*, IARC Scientific Publications No. 82. IARC: Lyon.

BRUNING PF, BONFRER JMG AND VERSTRAETEN AA. (1987). Prolactin levels after pregnancy. *N. Engl. J. Med.*, **317**, 384-385.

BRUZZI P, NEGRI E, LA VECCHIA C, DECARLI A, PALLI D, PARAZZINI F AND ROSSELI DEL TURCO M. (1988). Short term increase in risk of breast cancer after full term pregnancy. *B. Med. J.*, **297**, 1096-1098.

CUMMINGS P, STANFORD JL, DALING JR, WEISS NS AND McKNIGHT B. (1994). Risk of breast cancer in relation to the interval since last full term pregnancy. *Br. Med. J.*, **308**, 1672-1674.

HENDERSON BE AND BERNSTEIN L. (1991). The international variation in breast cancer rates: an epidemiological assessment. *Breast Cancer Res. Treat.*, **18**, S11-S17.

JANERICH DT. (1979). Pregnancy, breast cancer risk, and maternal-fetal genetics. *Lancet*, **1**, 327-328.

KALACHE A, MAGUIRE A, THOMPSON SG. (1993). Age at last full-term pregnancy and risk of breast cancer. *Lancet*, **341**, 33-36.

KVÅLE G AND HEUCH I. (1987). A prospective study of reproductive factors and breast cancer. II. Age at first and last birth. *Am. J. Epidemiol.*, **126**, 842-850.

LAMBE M, HSIEH C-C, TRICHOPOULOS D, EKBOM A, PAVIA M AND ADAMI H-O. (1994). Transient increase in the risk of breast cancer after giving birth. *N. Engl. J. Med.*, **331**, 5-9.

MILLER AB, BARCLAY THC, CHOI NW, GRACE MG, WALL C, PLANTE M, HOWE GR, CINADER B AND DAVIS FG. (1980). A study of cancer, parity and age at first pregnancy. *J. Chron. Dis.*, **33**, 595-605.

MUSEY VC, COLLINS DC, MUSEY PI, MARTINO-SALTZMAN D AND PREEDY JRK. (1987). Long-term effect of a first pregnancy on the secretion of prolactin. *N. Engl. J. Med.*, **316**, 229-234.

NEGRI E, LA VECCHIA C, DUFFY SW, BRUZZI P, PARAZZINI F AND DAY NE. (1990). Age at first and second births and breast cancer risk in biparous women. *Int. J. Cancer*, **45**, 428-430.

PIKE MC, KRAILO MD, HENDERSON BE, CASAGRANDE JT AND HOEL DG. (1983). 'Hormonal' risk factors, 'breast tissue age' and the age incidence of breast cancer. *Nature*, **303**, 767-770.

PRESTON DL, LUBIN JH, PIERCE DA AND McCONNEY ME. (1993). *EPICURE - Risk Regression and Data Analysis Software Manual*. Hirosoft International: Seattle.

ROSNER B, COLDITZ GA AND WILLETT WC. (1994). Reproductive risk factors in a prospective study of breast cancer: the nurses' health study. *Am. J. Epidemiol.*, **139**, 819-835.

RUSSO J, TAY LK AND RUSSO IH. (1982). Differentiation of the mammary gland and susceptibility to carcinogenesis. *Breast Cancer Res. Treat.*, **2**, 5-73.

RUSSO J, RIVERA R AND RUSSO IH. (1992). Influence of age and parity on the development of the human breast. *Breast Cancer Res. Treat.*, **23**, 211-218.

THOMAS DB. (1991). Rapporteur's report - epidemiology. *Breast Cancer Res. Treat.*, **18**, S31-S34.

VATTEN LJ AND KVINNSLAND S. (1992). Pregnancy-related factors and risk of breast cancer in a prospective study of 29 981 Norwegian women. *Eur. J. Cancer*, **28A**, 1148-1153.

WANG DY, DE STAVOLA BL, BULBROOK RD, ALLEN DS, KWA HG, VERSTRAETEN AA, MOORE JW, FENTIMAN IS, HAYWARD JL AND GRAVELLE IH. (1988). The permanent effect of reproductive events on blood prolactin levels and its relation to breast cancer risk: a population study of postmenopausal women. *Eur. J. Cancer Clin. Oncol.*, **24,** 1225–1231.

WILLIAMS EMI, JONES L, VESSEY MP AND McPHERSON K. (1990). Short term increase in risk of breast cancer associated with full term pregnancy. *Br. Med. J.*, **300,** 578–579.